# 3D bioprinting of prefabricated artificial skin with multicomponent hydrogel for skin and hair follicle regeneration

**DOI:** 10.7150/thno.104854

**Published:** 2025-02-10

**Authors:** Xiaoxiao Ma, Xiaohui Zhu, Sheng Lv, Chunyan Yang, Zihao Wang, Meilan Liao, Bohao Zhou, Yiming Zhang, Shiyu Sun, Ping Chen, Zhonghua Liu, Haiyan Chen

**Affiliations:** 1 The National and Local Joint Engineering Laboratory of Animal Peptide Drug Development, College of Life Sciences, Hunan Normal University, Changsha, 410081, People's Republic of China.; 2 East China Institute of Digital Medical Engineering, Shangrao, 334000, People's Republic of China.; 3 Peptide and Small Molecule Drug RD Platform, Furong laboratory, Hunan Normal University, Changsha, 410081, Hunan, People's Republic of China.

**Keywords:** 3D bioprinting, tissue engineering, wound healing, hair follicle, artificial skin

## Abstract

**Background:** The timely management of large-scale wounds and the regeneration of skin appendages constitute major clinical issues. The production of high-precision and customizable artificial skin via 3D bioprinting offers a feasible means to surmount the predicament, within which the selection of bioactive materials and seed cells is critical. This study is aimed at employing skin stem cells and multicomponent hydrogels to prefabricate artificial skin through 3D bioprinting, which enables the regeneration of skin and its appendages.

**Methods and Results:** We employed gelatin methacrylate (GelMA) and hyaluronic acid methacrylate (HAMA) as bioactive materials, in conjunction with epidermal stem cells (Epi-SCs) and skin-derived precursors (SKPs), to fabricate artificial skin utilizing 3D bioprinting. The photosensitive multicomponent hydrogel, comprising 5% GelMA and 0.5% HAMA, demonstrated excellent printability, suitable solubility and swelling rates, as well as stable mechanical properties. Moreover, this hydrogel exhibited exceptional biocompatibility, effectively facilitating the proliferation of SKPs while maintaining the cellular characteristics of both SKPs and Epi-SCs. The transplantation of this artificial skin into cutaneous wounds in nude mice led to complete wound healing and functional tissue regeneration. The regenerated tissue comprised epidermis, dermis, hair follicles, blood vessels, and sebaceous glands, closely resembling native skin. Remarkably, the artificial skin demonstrated sustained tissue regeneration capacity even after 12 h of *in vitro* culture, facilitating comprehensive functional skin regeneration.

**Conclusions:** Our research presented a skin repair strategy for prefabricated cell-loaded artificial skin, thereby successfully facilitating the regeneration of the epidermis, dermis, hair follicles, blood vessels, and sebaceous glands within the wound.

## Introduction

The skin is one of the most essential organs in the human body, constituting a continuous outer barrier system in conjunction with sweat glands, sebaceous glands, hair follicles (HFs), and other appendages 1. It performs several critical biological functions, including the resistance to foreign body invasion, regulation of body temperature, and prevention of water loss. Wounds arise from the compromise of skin integrity due to external trauma factors, including surgical procedures, thermal injury, electrical burns, and pressure-related injuries encountered in daily life 2. These wounds can result in pain, anxiety, infection, and even mortality, significantly impairing patients' quality of life while imposing a considerable burden on the healthcare system 3, 4. Based on pertinent retrospective analyses, the global advanced wound care market is projected to reach $18.7 billion by 2027 5, 6. Consequently, the identification of effective and rapid treatment options to enhance wound healing has become an urgent clinical challenge that requires immediate attention.

Current treatment modalities for extensive cutaneous wounds primarily encompass autologous skin transplantation, artificial skin substitutes, and cellular therapies 7. Autologous skin grafting remains the gold standard for the management of extensive cutaneous wounds; however, it is associated with several limitations, including secondary pain, limited availability of donor sites, and an increased risk of infection 8. Artificial skin substitutes are engineered to enhance wound healing by incorporating cells or extracellular matrices, thereby creating tissue-engineered bionic skin. For example, Biobrane consists of a double-layer nylon mesh infused with porcine type I collagen and coated with a silicone sheet, enabling it to function as a temporary covering for burns, skin graft donor sites, and hidradenitis suppurativa 9-11. Dermagraft, on the other hand, incorporates human neonatal foreskin fibroblasts onto an absorbable polylactic acid and polyglycolic acid mesh scaffold, rendering it suitable for the treatment of full-thickness diabetic foot ulcers 12-14. Nevertheless, the current skin substitutes utilized in clinical practice primarily fulfill a fundamental role in accelerating wound healing and have yet to achieve the objective of fully functional, scar-free skin regeneration that encompasses skin appendages such as HFs, sweat glands, blood vessels, and sebaceous glands.

As a vital appendage of the skin, HFs play a significant role in resisting external stimuli, establishing a protective barrier, and facilitating wound healing. Furthermore, they exert a considerable influence on the aesthetic appearance of the human body. Previous studies have demonstrated that dermal papilla cells (DPCs) possess the capacity for hair-inducing regeneration 15-17. Nevertheless, the utilization of DPCs in tissue engineering presents several limitations, including their relative scarcity and challenges in obtaining them *in vivo*, as well as difficulties in preserving their regenerative potential during *in vitro* culture. In contrast, skin-derived precursors (SKPs) are multipotent precursor cells located within the mammalian dermis, possessing the ability to differentiate into dermal, neural, and mesodermal cell lineages, thereby offering substantial potential for wound healing and HFs regeneration 18-21. Numerous studies have established that the interaction between epidermal stem cells (Epi-SCs) and SKPs is essential for the growth and developmental processes of skin appendages, including HFs, sebaceous glands, and nerves 22, 23. Consequently, the application of SKPs and Epi-SCs as seed cells for the development of tissue-engineered artificial skin may represent a promising strategy to enhance full-thickness wound healing.

Hydrogels are extensively utilized in tissue engineering owing to their remarkable water absorption capacity, moisturizing properties, biocompatibility, and three-dimensional porous architecture 24. Currently, hydrogel materials such as collagen, Matrigel, and alginate are employed in the fabrication of artificial skin 25. A single hydrogel may not suffice to fulfill the complex requirements of 3D bioprinting for the fabrication of artificial skin; therefore, composite hydrogels composed of a mixture of multiple materials represent a promising alternative. Gelatin methacrylate (GelMA) is synthesized from gelatin and methacrylic anhydride (MA), while hyaluronic acid methacrylate (HAMA) is derived from hyaluronic acid and MA. Both materials can be rapidly polymerized upon light exposure to form three-dimensional structures that facilitate cell growth and differentiation. In comparison to collagen and fibrin, GelMA exhibits enhanced biocompatibility while demonstrating relatively lower mechanical strength 26. In contrast, HAMA demonstrates lower biocompatibility but possesses superior mechanical strength and stability at the same concentration 27. Consequently, the multicomponent hydrogel comprising GelMA and HAMA offers advantages such as high biocompatibility, enhanced mechanical strength, a straightforward curing process, and excellent printability, making it suitable for the fabrication of artificial skin via 3D bioprinting.

In this study, we incorporated SKPs and Epi-SCs into multicomponent hydrogel composed of GelMA and HAMA, subsequently fabricating artificial skin utilizing 3D bioprinting technology. Cytological analyses revealed that the artificial skin not only significantly enhanced the stemness and HFs regeneration capabilities of SKPs, but also maintained the cellular characteristics of Epi-SCs. The artificial skin was implanted into the wounds of mice to promote complete skin regeneration, encompassing the epidermis, dermis, HFs, blood vessels, and sebaceous glands. Moreover, we conducted an evaluation of the preformed potential of the artificial skin and determined that it retained its biological regenerative capacity even after 12 h of *in vitro* culture. This research provides promising solutions to the existing challenges in skin and HFs regeneration, while also establishing a theoretical framework for future* in vitro* culture and preservation of artificial skin.

## Materials and methods

### Configuration of multicomponent hydrogels

Quantitative measurements of GelMA (Engineering For Life, China), HAMA (Engineering For Life, China), and Phenyl-2,4,6-trimethyl-benzoyl phosphate lithium (LAP, Advanced BioMatrix, USA) were conducted as detailed in Table 1. Subsequently, 5 mL of PBS (Gibco, USA) was added to each tube, and the mixture was dissolved at 60 ℃ for 30 min, with stirring every 10 min. Each multicomponent hydrogel was pasteurized by rapid cooling to 4 °C after being held at 75 °C for 30 min in the dark. This process was repeated for 3 to 5 cycles. All multicomponent hydrogels were prepared at a two-fold concentration, and subsequent experiments were conducted following a half-dilution.

### Scanning electron microscopy (SEM) analysis

Multicomponent hydrogels were cross-linked using UV light, rapidly frozen in liquid nitrogen, and subsequently freeze-dried. The dried samples were then placed on a sample plate coated with conductive adhesive, followed by the application of a thin layer of gold to their surfaces before being analyzed using scanning electron microscopy (Zeiss, Germany).

### Swelling rate assay

Multicomponent hydrogels were cross-linked using UV light. Each sample was weighed and subsequently immersed in a 12-well plate containing PBS. Samples were removed at various time intervals, and surface water stains were dried prior to reweighing. The swelling rate (M_S_) at each time point was calculated using the following formula, where M_0_ represents the initial weight and M_t_ denotes the weight after swelling at the specified time point.



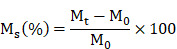



### Solubility assay

Multicomponent hydrogels were cross-linked using UV light. Each sample was freeze-dried, weighed, and subsequently immersed in a 12-well plate containing PBS. Following incubation for varying time intervals, the samples were extracted from the solution, surface moisture was removed, and the samples were then freeze-dried and reweighed. The dissolution percentage (M_T_) at each time point was calculated using the following formula, where M_0_ represents the initial dry weight and M_t_ denotes the dry weight of the material at the specified time point.



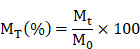



### Rheometry assay

The storage modulus (G') and loss modulus (G'') of multicomponent hydrogels were measured using the frequency sweep mode of a rotational rheometer (Thermo Fisher Scientific, USA). Following UV cross-linking of the multicomponent hydrogels, the shear strain was maintained at 1%, the temperature was set to 37 ℃, and a shear frequency scan was performed over the range of 0.1-10 rad/s.

### Printability assay

Initially, a three-dimensional CAD system (SolidWorks, USA) was utilized for 3D modeling, followed by the application of bioprinting software (Medprint Biotech, China) to optimize the printing parameters. Specifically, the multicomponent hydrogel solution was transferred into a 1 mL syringe, stored in the dark at 4 ℃ for 3 min, and subsequently printed using a 3D bioprinting machine (Livprint Norm, Medprin, China). During the inspection process, the printed model was designed as a square with a length of 15 mm, a thickness of 6 mm, and an infill density of 6%. After several pre-experimental parameter adjustments using 10% gelatin (Aladdin, USA), the optimal printing conditions were determined to be a nozzle diameter of 0.26 mm, a printing platform temperature of 6 °C, and a scanning speed of 8 mm/s. Following the printing process, UV light was employed to rapidly solidify the structure. Optical images were captured using a stereoscope (Olympus, Japan) and analyzed with Image-J software to determine the perimeter and area of the interconnected channels. This analysis enabled the calculation of the quantitative integrity of the multilayer structure, as previously described 28. The calculation method for the printability (P_r_) value is outlined in the following formula, where L denotes the perimeter of the enclosed area of the grille and A represents its area.



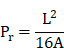



### Isolation and culture of SKPs and Epi-SCs

The isolation and culture of Epi-SCs and SKPs were conducted in accordance with the methods described in previous studies 23, 29, 30. The dorsal skin of C57BL/6J mice aged 0-3 days was excised and sectioned into pieces measuring 2-3 mm². The samples were treated with 0.3% Dispase II (Sigma-Aldrich, USA) for 60 min at 37 ℃ to manually separate the dermal and epidermal tissues. The dermal tissue was subsequently fragmented and treated with 0.4% collagenase I (Sigma-Aldrich, USA) for 90 min at 37 ℃ until a homogeneous suspension was achieved, which was then filtered through an 80-mesh sieve and centrifuged to isolate the SKPs at the bottom. SKPs were cultured in Dulbecco's Modified Eagle Medium (DMEM)/F12 (3:1, Gibco, USA), supplemented with 2% B27 (Gibco, USA), 20 ng/mL epidermal growth factor (EGF, Peprotech, USA), and 40 ng/mL basic fibroblast growth factor (bFGF, Peprotech, USA), while maintaining a cell density of 2-3×10⁵ cells/mL. Cytokines were replenished every 3 days, and the cells were passaged following digestion with TrypLE™ Express enzyme (Gibco, USA) on the seventh day. The epidermal tissue was excised, treated with 0.04% collagenase I at 37 ℃ for 60 min, filtered through a 100-mesh filter, and centrifuged to collect Epi-SCs from the sediment. Epi-SCs were cultured in Keratinocyte-SFM Epidermal Keratinocyte Medium (Gibco, USA) at a cell density of 1-2×10⁵ cells/mL, with medium changes performed every 2 days. Once the cells achieved confluence over a substantial area, they were digested using Accutase (Gibco, USA) and subsequently passaged.

### Cell proliferation assay

The Alamar Blue Kit (YEASEN, China) was utilized in accordance with the manufacturer's protocol to assess the proliferation of SKPs cultured in multicomponent hydrogels following 3D bioprinting. The SKPs cell suspension was combined with each multicomponent hydrogel (1:1) for 3D bioprinting, and subsequently placed in a 24-well plate for culture. For the detection assay, 300 μL of the Alamar Blue working solution (Alamar Blue: fresh culture medium = 1:9) was added to each well, followed by an incubation period of 4 h at 37 ℃ under standard cell culture conditions. Subsequently, 100 μL of the supernatant from each sample was carefully transferred to a new 96-well plate. The remaining alamar blue was aspirated, and the wells were washed with PBS. Following this, 300 μL of fresh culture medium was added to each sample to continue the culturing process. The optical density (OD) values of the supernatant were then measured at wavelengths of 570 and 630 nm using a microplate reader (BIOTEK, USA). The reduction rate was calculated, and the OD values of all groups were normalized according to the provided instructions.

### Cell viability assay

The viability of SKPs in multicomponent hydrogels after 3D bioprinting was assessed using a live/dead assay (KeyGEN BioTECH, China) in accordance with the manufacturer's instructions. SKPs were inoculated into multicomponent hydrogels for 3D bioprinting and subsequently cultured in 24-well plates. For the staining procedure, the original culture medium was first aspirated from the sample wells, followed by washing with PBS. Subsequently, 300 µL of the staining working solution (PBS:Calcein-AM:PI = 1000:1:1) was added, and the samples were incubated in the dark at 37 ℃ for 10 min prior to rinsing with PBS. Following the removal of excess dye, the samples were examined using a laser confocal microscope (Nikon, Japan), and subsequent quantitative analysis of the fluorescence images was conducted utilizing Image-J software.

### Alkaline phosphatase staining

An alkaline phosphatase (AP) staining kit was utilized to assess the expression of AP in SKPs cultured in multicomponent hydrogels for 4 days following 3D bioprinting. Conventionally cultured SKPs were harvested and centrifuged at 1500 rpm for 5 min to promote adherence to glass slides, after which AP staining was performed. SKPs embedded in multicomponent hydrogels following 3D bioprinting were cultured and subsequently stained in 24-well plates. The samples were fixed in 4% paraformaldehyde (PFA) at RT for 10 min and subsequently washed with PBS. 5-bromo-4-chloro-3-indolylphosphate (BCIP) and nitro blue tetrazolium (NBT) solutions were added, and the samples were incubated at room temperature in the dark for 4 h. Following washing with phosphate-buffered saline (PBS), the samples were examined using an optical inverted microscope (Olympus, Japan).

### RNA isolation and quantitative Real time PCR (RT-qPCR) analysis

This study investigated the gene expression levels associated with stemness and hair-inducing capabilities of SKPs in artificial skin using RT-qPCR. Total RNA was isolated and purified using a total RNA extraction kit (Takara, Japan). The quantity and purity of each RNA sample were assessed using a NanoDrop spectrophotometer (Thermo Scientific, USA). RNA was reverse transcribed into cDNA using the PrimerScript^TM^ RT Kit with gDNA Eraser (Takara, Japan). The RT-qPCR reaction was performed using SYBR® Green (Takara, Japan) on the Gentier 96E/96R system (Tianlong, China). The thermal cycling conditions were established at 95 ℃ for 45 s, followed by 95 ℃ for 5 s and 61 ℃ for 34 s, repeated for a total of 40 cycles. The primers used for mouse gene amplification in this study are detailed in Table 2. Glyceraldehyde 3-phosphate dehydrogenase (GAPDH) served as the internal reference, and the relative expression of the target gene was calculated using the ΔΔCt method.

### Flow cytometry analysis

Flow cytometry was utilized to evaluate the expression of specific markers in Epi-SCs. The artificial skin was cultured in a 24-well plate for 2 days, after which a mixed lysis solution (comprising 1 mg/mL hyaluronidase and 0.3 mg/mL GelMA lysis solution) was utilized to release the cells for flow cytometric analysis. In the conventional culture group, Epi-SCs were cultured in adherent dishes following digestion, and the cells were collected for flow cytometric analysis after 2 days. The samples were washed once with PBS and resuspended in 1% BSA (Aladdin, USA). A total of 100 μL of cell suspension, with a density exceeding 10^6^ cells/mL, was incubated with various fluorescently conjugated antibodies, including anti-CD29-FITC (1:25, BioLegend, USA) and anti-CD49f-PE (1:25, BioLegend, USA), or the control isotype IgG for 30 min at 4 ℃ in the dark. Following the incubation period, 1 mL of binding buffer was added, and the cell samples were analyzed using a flow cytometer (Beckman, USA), with data processed by CellQuest software.

### Artificial skin for *in vivo* skin regeneration

C57BL/6J mice (6-week-old, female/male) and BALB/c-nu/nu mice were purchased from Slac & Jingda Corporation of laboratory animals, Changsha, China. BALB/c-nu/nu mice were anesthetized with sodium pentobarbital (50 mg/kg), and a skin biopsy instrument with a 5 mm diameter was employed to create a symmetrical full-thickness skin defect on the dorsal surface. We utilized 5% GelMA-0.5% HAMA as biomaterials for 3D printing in the fabrication of artificial skin. The artificial skin was designed as a square with a side length of 5 mm, a thickness of 6 mm, and a filling density of 8%, incorporating Epi-SCs (5×10^7^ cells/mL) and SKP (1×10^8^ cells/mL). The constructs were cultured using CnT-Prime 3D Barrier medium (CellnTec, Switzerland). After culturing for 0, 6, and 12 h, the transplants were positioned onto the wound surface and secured with a transparent dressing (3M) and a self-adhesive elastic bandage. After 4 weeks, the mice were sacrificed, and the number of hairs was counted using dissecting microscope (Olympus, Japan). Furthermore, wound tissue samples were obtained for histological analysis. Throughout the experiment, all animals were housed in a temperature-controlled environment (20±1 ℃) with ad libitum access to food and water. All animal experiments conducted in this study were approved by the Animal Ethics Committee of Hunan Normal University and complied with the National Institutes of Health Guidelines for the Care and Use of Laboratory Animals.

### Immunofluorescence (IF) staining

Freshly regenerated mouse skin samples were collected, fixed in 4% PFA overnight, and subsequently washed with PBS for 12 h to remove excess PFA. Gradients of 10%, 20%, and 30% sucrose were subsequently applied for dehydration over a period of 12 h. The samples were embedded in tissue freezing medium (SAKURA Tissue-Tek® OCT Compound, USA) and stored at -80 ℃. Cell samples were fixed in 1% PFA for 10 min. Frozen tissue sections of skin and SKPs samples were incubated overnight at 4 ℃ with various primary antibodies: anti-nestin (1:50, Abcam, UK), anti-fibronectin (1:50, GeneTex, USA), anti-BMP6 (1:50, Abcam, UK), anti-CD31 (1:30, GeneTex, USA), anti-biotin (1:50, eBioscience, USA), anti-keratin 14 (K14, 1:50, BioLegend, USA), and anti-Keratin 1 (K1, 1:50, BioLegend, USA). Samples were washed with PBS and incubated with TRITC/cy3 or FITC-conjugated secondary antibodies for 2 h at RT. Subsequently, the nuclei were stained with 4',6-diamidino-2-phenylindole (DAPI) for 15 min at RT. Following the removal of excess dye with PBS, the samples were mounted and visualized using a confocal microscope.

### Hematoxylin and eosin (H&E) staining

Freshly regenerated skin tissue was fixed in 4% PFA at RT for a duration of 12-24 h. Subsequently, the specimens were dehydrated using a gradient of ethanol concentrations: 70%, 80%, 90%, 95%, and 100%, with each concentration applied for a duration of 90 min. Following dehydration, the specimens were embedded in paraffin wax. The paraffin-embedded tissue sections were subsequently rehydrated sequentially with 100% ethanol, 95% ethanol, 75% ethanol, and deionized water, with each rehydration step lasting for 3 min. H&E staining was performed, in which the nuclei were stained with hematoxylin and the cytoplasm was counterstained with eosin. Finally, the slides were mounted and examined using an optical inverted microscope (Olympus, Japan).

### Statistical analysis

All experiments were conducted a minimum of three times, and results are expressed as mean±s.e.m., unless otherwise specified. GraphPad Prism 8 software was employed for data visualization, and a Student's t-test was performed to assess statistical differences between the two groups. A probability (P) value<0.05 was considered statistically significant. Asterisks and letters were respectively utilized to indicate significance between two groups and among multiple groups.

## Results

### Characterization of multicomponent hydrogels and their 3D bioprinting

**Figure 1:**
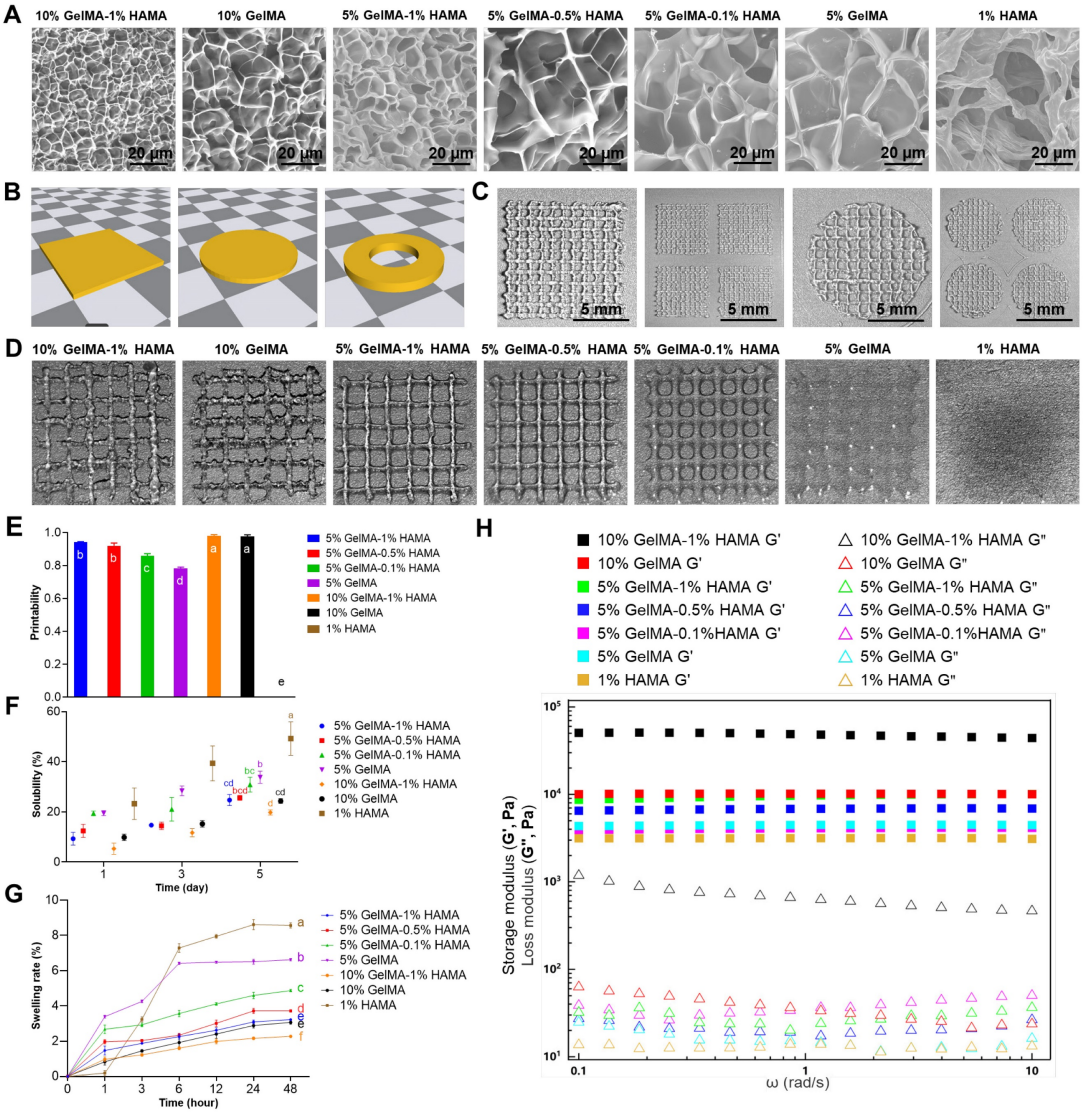
Characterization and evaluation of printability of multicomponent hydrogels. (A) SEM images of multicomponent hydrogels at varying concentrations (Scale bar: 20 μm). (B) Fabrication of 3D bioprinting models. (C) Implementation of layer-by-layer grid 3D bioprinting (Scale bar: 5 mm). (D) Macroscopic images of 3D bioprinting of various multicomponent hydrogel. (E) Quantification of the Pr value for each multicomponent hydrogel. There were significant differences between groups labeled with different letters, but no significant differences between groups containing the same letter. (F) Evaluation of solubility in various multicomponent hydrogels. There were significant differences between groups labeled with different letters, but no significant differences between groups containing the same letter. (G) Assessment of swelling rates in various multicomponent hydrogels. There were significant differences between groups labeled with different letters, but no significant differences between groups containing the same letter. (H) Evaluation of rheological properties in various multicomponent hydrogels.

Biomaterials play a pivotal role in the fabrication of tissue-engineered artificial skin; therefore, we initially optimized the multicomponent hydrogel concentration. The tunable pore size and microstructure of hydrogels enable the fabrication of engineered tissues that closely mimic the structures and functions of natural tissues 31. SEM analysis indicated that multicomponent hydrogels at varying concentrations exhibited an interconnected three-dimensional porous network structure, with pore sizes ranging from 5 to 30 μm, which progressively decreased as hydrogel concentration increased (Figure 1A). Swelling and dissolution performance can be employed to evaluate the structural integrity and stability of tissue-engineered artificial skin within the body, which are essential for the development of optimal artificial skin 32-35. The results of the solubility and swelling tests indicated that as the concentration of the hydrogel increases, both the swelling capacity and solubility of the multicomponent hydrogel decrease progressively (Figure 1F, G). Furthermore, when hydrogel materials are utilized at trauma sites within the human body, a requisite level of mechanical strength is necessary to withstand deformation induced by daily activities. Rheological testing results demonstrated that, within the scanning frequency range of 0.1 to 10 rad/s, the G' of each multicomponent hydrogel significantly exceeds the G'', thereby confirming its capacity to maintain a stable solid elastic structure (Figure 1H). Moreover, the mechanical stability of multicomponent hydrogels exhibits a positive correlation with the concentration of the multicomponent hydrogel. This correlation may arise from the increased charge density and polymer concentration within the hydrogel system as concentrations escalate, subsequently enhancing the storage modulus of the hydrogel.

To enable large-scale production and application of artificial skin, we evaluated the 3D printability of various multicomponent hydrogels. Employing SolidWorks software, we developed several printing models that were subsequently utilized in a 3D bioprinting machine to execute layer-by-layer grid printing (Figure 1B, C). Subsequently, various multicomponent hydrogels were employed for the 3D bioprinting of square grids. Quantitative analysis of macroscopic printing images and Pr values demonstrated that as hydrogel concentration increased, the models exhibited enhanced regularity (Fig. 1D, E). Notably, the hydrogel with a 1% HAMA concentration resulted in complete fusion of the printed grid scaffold lines due to excessive liquefaction. In contrast, multicomponent hydrogels containing 10% GelMA-1% HAMA, 10% GelMA, and 5% GelMA-1% HAMA exhibited higher Pr values; however, excessive gelation compromised the performance of the printed models, leading to issues such as bending, stacking, or even breaking in certain areas.

### SKPs viability, proliferation, and AP expression in multicomponent hydrogels

**Figure 2:**
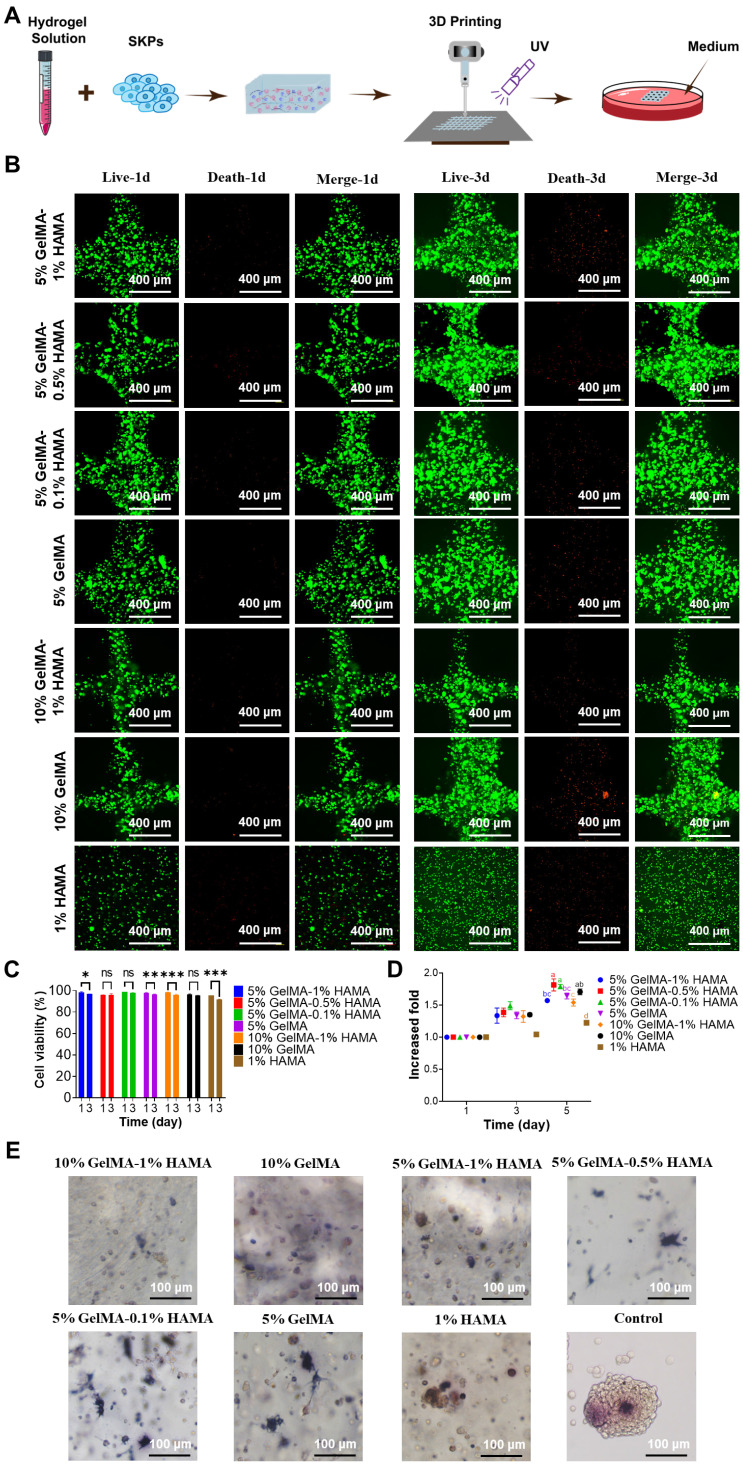
Proliferation and viability of SKPs in multicomponent hydrogels. (A) Schematic representation of the 3D bioprinting process for multicomponent hydrogels incorporated with SKPs. (B) Live/dead staining of SKPs within multicomponent hydrogels after 1 and 3 days of culture. (Scale bar: 400 μm). (C) Quantification of cellular viability. Where “ns” denotes no significant difference, “*” represents a P value less than 0.05, “**” stands for a P value less than 0.01, and “***” indicates a P value less than 0.001. (D) Proliferation of SKPs within multicomponent hydrogels after 1 and 3 days of culture. There were significant differences between groups labeled with different letters, but no significant differences between groups containing the same letter. (E) AP staining images of SKPs cultured in multicomponent hydrogels for 4 days (Scale bar: 100 μm).

To investigate the effects of the 3D bioprinting process and various multicomponent hydrogels on the proliferation and activity of SKPs, SKPs were combined with different multicomponent hydrogels, followed by 3D bioprinting and subsequent culture in 24-well plates (Figure 2A). The proliferation and activity of SKPs were evaluated on day 1 and day 3, respectively. The results of cell viability staining revealed that on day 1 of culture following 3D bioprinting, the activity of SKPs within the multicomponent hydrogels remained significantly high, exceeding 95%, thereby indicating that the 3D bioprinting process did not induce any apparent cellular damage (Figure 2B, C). After 3 days of culture, we observed that SKPs proliferated to varying extents across each group of multicomponent hydrogels. Although cell viability was slightly diminished, it remained above 90%, indicating that the multicomponent hydrogels demonstrated favorable biocompatibility. The results of the cell proliferation assay further demonstrated that SKPs exhibited varying degrees of proliferation across each multicomponent hydrogel (Figure 2D).

Alkaline phosphatase (AP) is highly expressed in various types of stem cells, including pluripotent stem cells, embryonic stem cells, dermal stem cells, and neural stem cells 36, 37. Previous studies have shown that the expression level of AP is closely correlated with the hair-inducing capability of DPCs 38. Consequently, we conducted AP staining on SKPs cultured within multicomponent hydrogels. The staining results demonstrated that SKPs within each multicomponent hydrogel exhibited significant expression of AP, indicating that these biomaterials did not adversely affect the HFs regeneration potential of SKPs (Figure 2E). Notably, the expression level of AP in SKPs was significantly elevated within multicomponent hydrogels (5% GelMA, 5% GelMA-0.1% HAMA, and 5% GelMA-0.5% HAMA), where cellular extension morphology was observed.

Upon comprehensive evaluation of the results, it was concluded that the 5% GelMA-0.5% HAMA concentration exhibited favorable swelling and dissolution characteristics, excellent 3D bioprinting performance, and stable rheological properties. Furthermore, the multicomponent hydrogel at this concentration effectively maintained the viability and proliferation of SKPs, supported their morphological expansion, and promoted high expression levels of AP. Consequently, 5% GelMA-0.5% HAMA was selected as the biomaterial for 3D bioprinting artificial skin in subsequent research.

### Cytological analysis of Epi-SCs and SKPs in artificial skin

**Figure 3:**
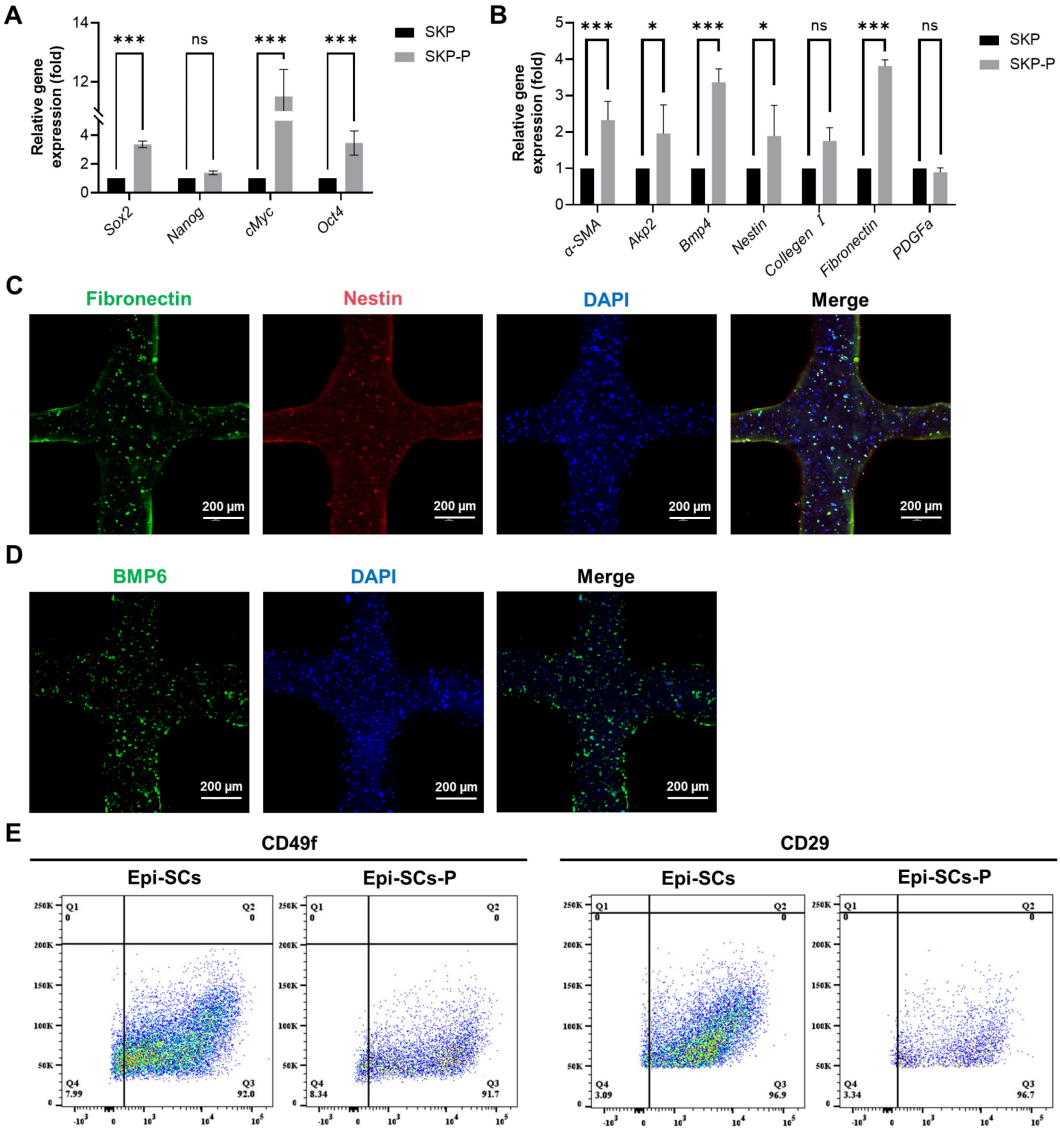
Cytological analysis of stem cells in artificial skin. (A and B) RT-qPCR was employed to assess the expression of stemness and hair-inducing potential in SKPs cultured for 3 days within the artificial skin. SKP embodies the conventional culture group, whilst SKP-P denotes the three-dimensional culture group of artificial skin. Where “ns” denotes no significant difference, “*” represents a P value less than 0.05, “**” stands for a P value less than 0.01, and “***” indicates a P value less than 0.001. (C and D) Representative immunofluorescence staining images demonstrated a high expression of specific proteins, including BMP6, nestin, and fibronectin, in SKPs located within an artificial skin environment. (Scale bar: 200 μm). (E) Flow cytometry analysis of CD29 and CD49f expression levels in Epi-SCs from conventional culture and artificial skin. Epi-SC represents the conventional culture group, whereas Epi-SC-P designates the three-dimensional culture group of artificial skin.

In order to evaluate the impact of 3D bioprinting and three-dimensional culture on SKPs, the cells were cultured for a period of 3 days, during which cytological changes were observed. IF results demonstrated that SKPs stably expressed the marker proteins Nestin, Fibronectin, and BMP6 in artificial skin, indicating that this environment effectively preserves the cellular characteristics of SKPs (Figure 3C, D). We further assessed the effects of multicomponent hydrogels on the stemness and hair-inducing potential of SKPs using RT-qPCR. The results demonstrated a significant increase in the expression of stemness genes, including *octamer-binding transcription factor 4* (*Oct4*), *SRY-box 2* (*Sox2*), and *c-Myc* (Figure 3A). Among the genes implicated in hair-inducing capacity, the expression levels of *α-smooth muscle actin* (*α-SMA*), *bone morphogenetic protein 4* (*BMP4*),* alkaline phosphatase 2* (*Akp2*), *Nestin*, and *fibronectin* were significantly elevated, whereas the expression levels of *collagen I* and *platelet-derived growth factor-α (PDGFα)* exhibited slight increases that were not statistically significant (Figure 3B). These results further demonstrated that the artificial skin we prepared had significant potential for HFs regeneration. Epi-SCs play a crucial role in wound healing by interacting with SKPs, which is significant for sebaceous gland and epidermal regeneration. Analysis of flow cytometry results revealed that, although the expression levels of CD49f and CD29 in Epi-SCs cultured with multicomponent hydrogel were slightly diminished compared to those in the conventional culture group, no statistically significant difference was observed (P>0.05) (Figure 3E). These findings indicated that the artificial skin developed in this study possesses the potential to regenerate skin appendages.

### 3D bioprinting of artificial skin incorporating SKPs and Epi-SCs promotes the regeneration of skin and HFs *in vivo*

**Figure 4:**
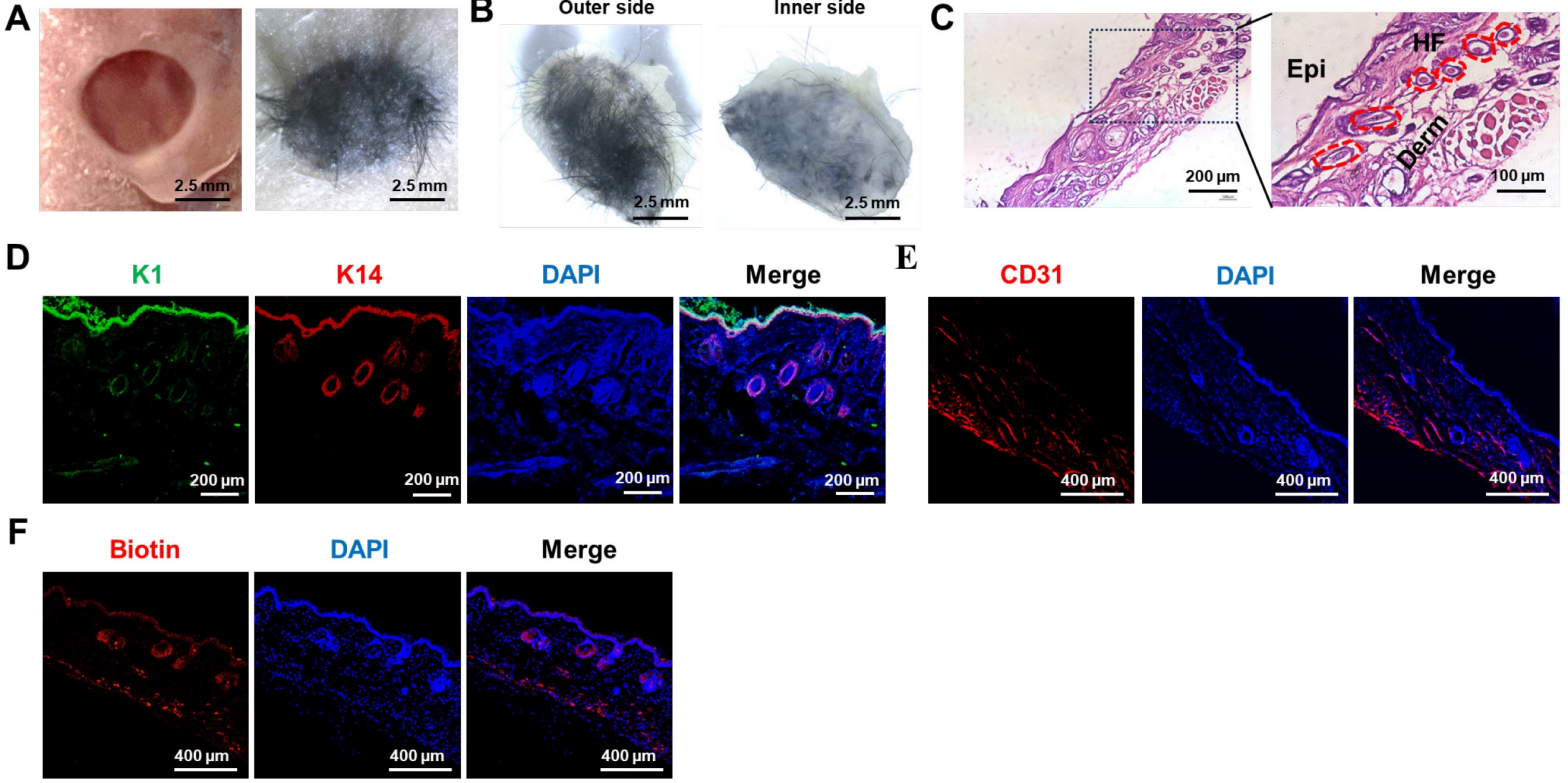
The artificial skin exhibited the ability to regenerate both the skin and its appendages. (A) Significant hair growth was observed 4 weeks after the transplantation of the artificial skin (Scale bar: 2.5 mm). (B) Representative images depicted both the outer and inner surfaces of the regenerated tissue four weeks post-grafting of the artificial skin (Scale bar: 2.5 mm). (C) H&E staining revealed the structural characteristics of the regenerated tissue (Scale bar: 200 μm and 100 μm). (D) IF staining for K1 and K14 indicated lamellar epidermal regeneration within the tissue (Scale bar: 200 μm). (E) IF staining for CD31 indicated vascular regeneration within the regenerating tissue (Scale bar: 400 μm). (F) IF staining for biotin suggested the regeneration of sebaceous glands within the regenerated tissue (Scale bar: 400 μm).

The efficacy of the artificial skin was further evaluated using models for skin and hair follicle reconstruction. Initially, a circular full-thickness skin biopsy punch was utilized to create a 5 mm diameter wound on the dorsal surface of a nude mouse. Subsequently, artificial skin was fabricated via 3D bioprinting using a multicomponent hydrogel comprising Epi-SCs, SKPs, and 5% GelMA-0.5% HAMA, which was then applied to the wound (Figure 4A). After a period of 4 weeks, we observed that the artificial skin facilitated complete wound healing, accompanied by notable hair growth (Figure 4B). H&E staining confirmed the regeneration of HFs and the formation of both epidermis and dermis within the wound (Fig. 4C). To further investigate the epidermal architecture of the regenerated skin tissue, co-staining for keratin 1 (K1) and keratin 14 (K14) immunofluorescence was conducted. K1 is expressed in differentiated keratinocytes, while K14 is predominantly expressed in Epi-SCs 39. The results demonstrated that the artificial skin facilitated the regeneration of a lamellar epidermis that closely resembles natural skin (Figure 4D). At present, a major limitation of artificial skin is its inability to regenerate skin appendages.

Consequently, skin appendages were assessed in the regenerated skin tissue. Considering the essential role of blood vessels in organ regeneration, particularly in the transport of oxygen and nutrients, we assessed the presence of blood vessels in the regenerated skin tissue using CD31 IF staining, as CD31 serves as a marker for angiogenic endothelial cells 40. The results confirmed that the artificial skin developed in this study successfully achieved vascular regeneration of the skin (Figure 4E). Additionally, biotin, a specific product of sebaceous glands, exhibited significant expression in the regenerated skin as indicated by IF staining, suggesting the regeneration of sebaceous glands (Fig. 4F) 41. These results indicated that the study had established a promising protocol for the *in vivo* regeneration of skin, HFs, and other skin appendages.

### Prefabricated artificial skin facilitates the regeneration of skin and HFs *in vivo*

**Figure 5:**
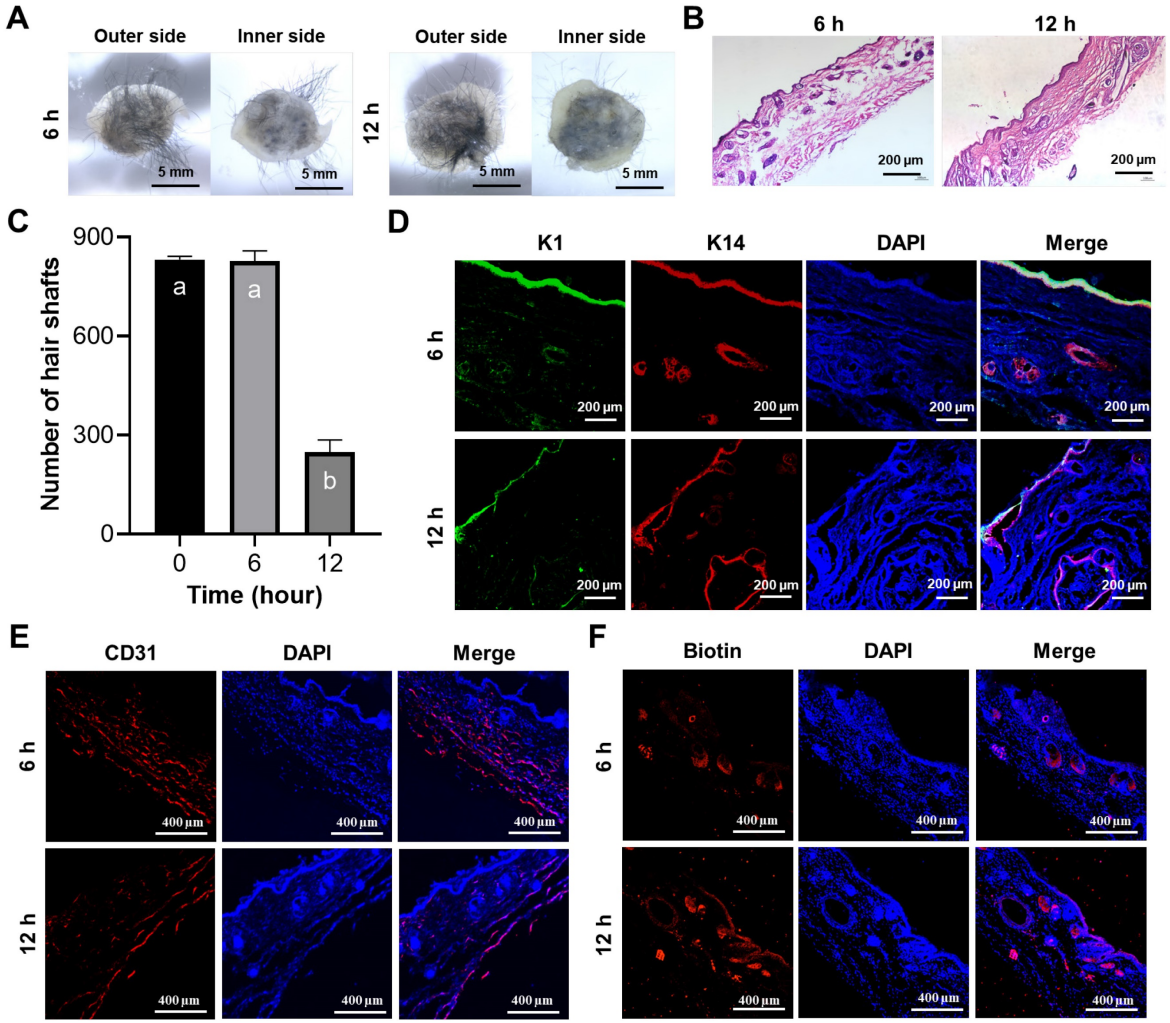
The prefabricated artificial skin exhibited the ability to regenerate both the epidermis and its associated appendages. (A) Representative images illustrated both the external and internal surfaces of the regenerated tissue after 4 weeks post-transplantation following the implantation of the prefabricated artificial skin (Scale bar: 5 mm). (B) H&E staining elucidated the structural characteristics of the regenerated tissue (Scale bar: 200 μm). (C) Statistical analysis of hair regeneration within the artificial skin was conducted. There were significant differences between groups labeled with different letters, but no significant differences between groups containing the same letter. (D) IF staining for K1 and K14 validated the epidermal characteristics of the regenerated tissue (Scale bar: 200 μm). (E) IF staining for CD31 demonstrated vascular regeneration within the regenerated tissue (scale bar: 400 μm). (F) IF staining for biotin illustrated the regeneration of sebaceous glands within the regenerated tissue (Scale bar: 400 μm).

In addition to the challenges associated with regenerating skin appendages using artificial skin currently under clinical investigation, the prefabrication of cell-laden artificial skin also poses significant difficulties. Should patients be able to prepare artificial skin prior to surgery and successfully regenerate skin appendages, the clinical applicability of such advancements would be substantially enhanced 42. To preliminarily investigate the prefabrication of artificial skin incorporating living cells, the artificial skin was cultured *in vitro* for 6 and 12 h prior to *in vivo* transplantation. After 4 weeks, we observed that the artificial skin continued to facilitate hair regeneration on the wound surface following 6 and 12 h of *in vitro* culture (Figure 5A). H&E staining further confirmed the regeneration of the epidermis, dermis, and HFs in the wound (Figure 5B). By quantifying the number of hairs in the regenerated skin, we observed that as the culture duration increased, the number of hairs gradually decreased; however, HFs regeneration remained feasible within 12 h (Figure 5C). Additionally, IF co-staining for K1 and K14 further confirmed that epidermal regeneration akin to that of natural skin was achieved (Figure 5D). IF staining for CD31 and biotin on the regenerated skin also demonstrated the regeneration of blood vessels and sebaceous glands (Figures 5E, F). These results demonstrated that the artificial skin we developed was capable of regenerating both the skin and its appendages within a 12-h period. No additional differences were observed in the effects of tissue regeneration, aside from a progressive reduction in hair density with extended culture time. Our research establishes a theoretical framework for the prefabrication of cell-laden artificial skin.

## Discussion

The process of skin wound repair encompasses the phases of inflammation and hemostasis, granulation tissue formation, and proliferative remodeling, which occur in a staggered and stepwise manner. Except for fetal wounds or superficial injuries that can heal without scarring, the healing process of extensive wounds in adults usually leads to scar formation. The mechanism of scar formation primarily encompasses the disorder in growth factor secretion, the augmentation of extracellular matrix, and the activation of fibroblasts 43. During the inflammatory and hemostatic phases, chemokines released within the wound recruit and activate inflammatory cells, which subsequently give rise to the activation of stromal fibrogenic effector cells, predominantly fibroblasts 44. Furthermore, excessive secretion of pro-inflammatory factors such as interleukin (IL)-1α, IL-1β, IL-6, and tumor necrosis factor (TNF)-α can promote chronic wound inflammation, thereby significantly increasing the likelihood of scar formation 45. During the proliferative remodeling phase, actin within fibroblasts forms microfilament bundles, and α-SMA is integrated into the microfilament bundles to further augment the traction force of the cells, with fibroblasts gradually differentiating into myofibroblasts 46. Myofibroblasts induce wound contraction and facilitate the maturation of granulation tissue, and their secretion of extracellular matrix (ECM) can partially restore the tensile resistance of skin tissue 47, 48. The relative quantities of ECM proteins that induce fibrosis may vary in different tissues, yet the principal types of proteins are essentially similar, such as type Ⅰ and type Ⅲ collagen, fibronectin, and basement membrane proteins 49. Myofibroblasts typically commence undergoing programmed cell death subsequent to wound healing, followed by a progressive reduction in number 50. However, if myofibroblasts remain abundant after complete wound healing, the continuous contractile action of these cells can result in tissue deformation, and the continuous production of ECM components can lead to excessive deposition of collagen, fibronectin, etc., causing severe scar formation 51. The regenerated scar tissue often lacks functional appendages and is susceptible to factors such as ultraviolet radiation, temperature fluctuations, and arid environments 52. The development of artificial skin capable of regenerating skin appendages has consistently been a central focus of research in bioengineering and biomedicine. The advancement of cell-laden 3D bioprinting technology presents significant potential for achieving fully functional skin regeneration and holds considerable promise for clinical applications.

During the wound healing process, the regeneration of HFs, blood vessels, and sebaceous glands poses significant challenges. In the field of tissue engineering, cells play a pivotal role in facilitating organ regeneration. Currently, mesenchymal stem cells (MSCs), DPCs, and induced pluripotent stem cells (iPSCs) are extensively employed for skin regeneration (Table 3). MSCs can proliferate extensively *in vitro* while maintaining the capacity to differentiate into both epidermal and dermal cell lineages. However, there are relatively few studies that investigate their role in the regeneration of skin appendages 53. DPCs, located at the base of hairs, serve as a central component that connects and regulates the entire population of HF cells. DPCs can modulate HF growth through a paracrine mechanism and play a pivotal role in the growth, development, and cycling of HFs 54. Research indicates that DPCs and their exosomes can stimulate HF growth by promoting the proliferation and migration of outer root sheath cells (ORSCs), while also regulating their cell cycle status 55. Osada *et al.* successfully induced the formation of new HFs in human skin through the application of DPCs cultured in three-dimensional microspheres 56. However, the limited availability of DPCs poses challenges in sustaining hair regeneration capabilities *in vitro*, thereby constraining their clinical applications. Furthermore, iPSCs exhibit similarities to embryonic stem cells in terms of cell morphology, gene expression profiles, protein expression, epigenetic modifications, and differentiation potential. Studies have demonstrated that iPSCs can be cultured and differentiated into skin with hair using the organoid system 57. However, iPSCs pose safety risks during the reprogramming process, and the technology remains immature, rendering it unsuitable for current clinical applications. SKPs exhibit gene expression patterns analogous to those of DPCs, including *nexin*, *Wnt5a*, and *versican*, and they demonstrate a comparable capacity for hair-inducing regeneration 58. Notably, the potential hair follicle regeneration effect of SKPs may signify their potential in scar-free wound healing. Some studies have indicated that the presence of HFs can markedly reduce scar formation during wound healing 59. Firstly, the high expression of BMP in HFs can mitigate the degree of fibrosis induced by TGF-β in multiple organs 60. Secondly, myofibroblasts, serving as the main effector cells in the formation of hypertrophic scars and keloids, are capable of differentiating into adipocytes via the BMP signaling pathway 61. Finally, it has been demonstrated that SKPs possess the capability to alleviate inflammation and facilitate angiogenesis during wound healing, which are also of great significance for promoting scar-free wound healing. As for Epi-SCs, studies have demonstrated that Epi-SCs play a crucial role in the regeneration of sebaceous and sweat glands through their interactions with SKPs 62. In addition, Epi-SCs are capable of achieving scar-free skin regeneration through secreting growth factors and remodeling the ECM during wound healing 63. Some studies have revealed that the quantity of Epi-SCs in hypertrophic scar tissue is significantly decreased and the differentiation behavior is disordered, which might result in the disorder of the skin epidermal structure and function and the reduced healing ability 64. Simultaneously, Epi-SCs has a unique epithelial mesenchymal transition effect and has also been associated with immunomodulation and anti-inflammation, which is important for promoting wound healing 65.

Hydrogel materials are widely employed in tissue engineering due to their three-dimensional porous structure, which promotes stem cell adhesion and facilitates the reconstruction of the microenvironment. The interaction between hydrogels and stem cells is highly complex, with multiple physical properties exerting key roles in regulating stem cell fate 100. Firstly, and most crucially, the pore size of the hydrogel, where cell signals from neighboring cells in the 3D hydrogel system may outweigh the matrix signals, thereby maintaining the stem cells in a quiescent state. Cell-cell interactions can regulate stem cell properties via the secretion of signaling molecules or direct contact 101. Studies have demonstrated that appropriate pore sizes can induce stem cells to differentiate into specific lineages, such as angiogenesis (50 to 150 μm), chondrogenesis (90 to 250 μm), and dermatogenesis (20 to 100 μm) 102, 103. Secondly, hydrogel stiffness also plays a significant role in regulating stem cell behavior in hydrogels. Studies have discovered that hydrogels with tissue-specific matrix stiffness can facilitate the differentiation of different types of stem cells 104. Specifically, it has been discovered that stem cells can differentiate into neurogenic cells in softer hydrogel materials, whereas they are prone to differentiate into osteogenic or myogenic cells in harder hydrogel materials 105. Subsequent studies have disclosed that cells might respond to different hydrogel stiffness and transduce mechanical signals through multiple signaling pathways, including RhoA, Rac, Cdc42, GTPases, and Hippo pathways 106. Thirdly, stem cells in hydrogels can also remodel ECM by secreting proteases to degrade the biomaterials and thus meet their needs 107. Most stem cells in degradable hydrogels demonstrate higher cell differentiation potential and express higher levels of cell markers. Nevertheless, the dynamic degradation of hydrogels is frequently accompanied by alterations in other properties, such as stiffness, swelling, and pore size, which directly or indirectly regulate cell fate 108. At present, the impact of different hydrogel degradation rates on cell function is not fully comprehended and requires further investigation.

In practical applications, whether utilizing natural hydrogels such as Matrigel, collagen, and alginate or chemically synthesized hydrogels like polyacrylamide, GelMA and HAMA, each type presents specific limitations when used in isolation. Single-component hydrogels frequently modify the intermolecular forces of the polymer through adjusting the hydrogel concentration, which subsequently alters the physical characterization of the hydrogel. However, in the multicomponent hydrogel, aside from the influence of concentration, the complex interaction among different components is also of crucial significance for the influence of physical properties, thereby making it more controllable. For instance, some studies have fabricated multicomponent hydrogels with abundant structural layers and balanced mechanical properties by adding a small quantity of polyvinylpyrrolidone (PVP) to polyvinyl alcohol (PVA) 109. Further structural research discovered that apart from the crystallization region formed among PVA chains, the multicomponent hydrogel also possessed various hydrogen bonds and covalent crosslinking between PVA and PVP, which led to superior performance. In this study, a multicomponent hydrogel suitable for the fabrication of cell-containing artificial skin through 3D bioprinting was acquired by adding a small quantity of HAMA hydrogel to the GelMA hydrogel. Among them, GelMA is derived from gelatin and is extensively utilized in skin tissue regeneration due to its numerous advantages such as high cytocompatibility, low antigenicity, and tissue adhesion 110. However, the mechanical properties of GelMA are inadequate, which can be enhanced by adding other biomaterials in clinical application. The addition of a small quantity of HAMA not only did not modify the properties of GelMA, but also significantly enhanced the mechanical properties. The main reason could be attributed to the electrostatic interaction between the protonated carboxyl group of HAMA and the free lysine group of GelMA. Additionally, hydrogen bonds and hydrophobic interactions among aldehyde, carboxyl, and amine groups within the polymer chain might also exert an effect 111. More significantly, HAMA is modified from hyaluronic acid, which has been demonstrated to induce the expression of hair follicle regrowth-related factors and hair follicle markers by stimulating cell contact and activating the Wnt pathway 112. In another study, the integration of low levels of HAMA into GelMA was capable of replicating the collagen and glycosaminoglycan composition in native skin and effectively facilitating the epithelial-mesenchymal interaction during hair follicle development *in vitro* by establishing appropriate intercellular contacts and signaling 113.

This study employed a photosensitive multicomponent hydrogel (5% GelMA-0.5% HAMA) as biomaterials in conjunction with Epi-SCs and SKPs to fabricate artificial skin utilizing 3D bioprinting technology. The multicomponent hydrogel displays outstanding structural properties, mechanical properties, and biocompatibility. Firstly, this concentration multicomponent hydrogel possesses a void structure of approximately 25 μm, which is in accordance with the previous results of hydrogel pores suitable for skin regeneration. Secondly, the hydrogel exhibits excellent mechanical properties. The rheological properties test indicated that its G' was significantly larger than G" at a certain vibration frequency, suggesting that the material has a stable elastic structure. The storage modulus can reflect the stiffness of the material to a certain extent. Currently, there are scarce studies regarding the effect of hydrogel stiffness on stem cell differentiation, and the range of hydrogel stiffness suitable for different tissue regeneration has not been discriminated in detail. However, existing studies concur that only hydrogels with a storage modulus greater than 1.6 kPa can not only induce stem cell adhesion leading to mechanical conduction but also simulate the specific physiological extracellular matrix (ECM) of stem cells and trigger stem cell differentiation 104, 114. The storage modulus of the multicomponent hydrogel material used in this study was measured to be 6.5 kPa, indicating that the material not only has a stable solid structure, but also can effectively affect cell behavior. Finally, the multicomponent hydrogel demonstrated only 4% swelling after 24 h and maintained approximately 70% structural integrity after 5 days, which has positive implications for clinical applications. Meanwhile, we selected a lattice structure with micrometer pore size as a printing model, which is not only conducive to judging the superior printability of the multicomponent hydrogel but also conducive to promoting the recycling of nutrients and metabolites 115. Meanwhile, the model can significantly reduce the consumption of cells and biomaterials, which can reduce clinical costs. However, according to previous studies, SKPs and Epi-SCs in artificial skin can spontaneously accumulate in the wound and participate in the formation of the dermis and epidermis. Therefore, the printed model can be adapted to different symptoms in practical applications 104. Moreover, the artificial skin not only preserved the cellular properties of SKPs and Epi-SCs, but also enhanced the stemness and hair-inducing ability of SKPs. This enhancement may be ascribed to the capacity of three-dimensional culture models to replicate the *in vivo* environment through bioactive materials, thereby offering a more physiologically relevant context to guide cellular behavior and enhance their functionality 116, 117. Further *in vivo* studies demonstrated that the artificial skin could achieve complete wound regeneration, with the regenerated tissue exhibiting characteristics of the epidermis, dermis, blood vessels, HFs, and sebaceous glands that closely resemble those of healthy skin.

The prompt application of artificial skin is essential for contemporary clinical treatments. The *in vivo* application of cell-laden artificial skin may result in the degradation of the scaffold and the subsequent release of cells, primarily due to high-density cellular activity or metabolism. This phenomenon constrains the progress of clinically prefabricated artificial skin. In this study, the engineered artificial skin was cultured *in vitro* for a maximum of 12 h prior to transplantation onto skin wounds. Notably, successful full-thickness skin healing was achieved, and the regenerated skin closely resembled normal skin, except for a reduced number of hairs. These findings present novel strategies for the fields of wound healing and HFs regeneration, as well as innovative approaches for the regeneration of skin appendages in extensive wounds. Furthermore, we establish a foundational basis for future research on the *in vitro* culture of cell-laden artificial skin.

The utilization of 3D bioprinting technology in combination with stem cells for the preparation of artificial skin holds significant potential in clinical application; however, certain challenges still exist. First of all, the verification of the efficacy of human skin stem cells is urgent, which poses a challenge for exploring the isolation and culture technology of mature human skin stem cells. Secondly, the large-scale culturing of stem cells has been a key issue restricting their clinical application. Although current three-dimensional culturing methods can effectively facilitate the proliferation of skin stem cells, they are still distant from engineering applications. Exploratory 3D bioprinting with various biomaterials, printed models, cell densities, and media is a potential solution. Additionally, effective breakthrough notions might be stem cell expansion via microfluidic technology or bioreactors. Finally, considering that SKPs and Epi-SCs exert crucial interactions during folliculogenesis, the utilization of these two cells for hair follicle organoid culture *in vitro* is a highly promising research orientation. A study had shown that dermal and epidermal stem cells were embedded on Matrigel to achieve skin and hair follicle regeneration, and tracer assays of both types of stem cells in regenerated tissues with fluorescent labelling revealed that dermal stem cells could differentiate into hair papillae and dermis, and epidermal stem cells into hair shafts and epidermis, during the skin regeneration process 118. In addition, previous studies have successfully fabricated hair follicle organoids that can achieve folliculogenesis and hair growth *in vitro* by using low concentrations of Matrigel to control the spatial arrangement of epithelial and mesenchymal cells, but these hair follicles were not transplanted into animals 119. Therefore, 3D bioprinting of SKPs and Epi-SCs with core-shell structure model or microsphere structure model and subsequent culturing in an appropriate induction manner might result in hair follicle formation. However, since the co-culture mode and directed induction protocol of SKPs and Epi-SCs remain unclear, the utilization of iPSCs or MSCs could be potential alternatives. As the demand for personalized treatment in the medical field continues to increase, it is imperative to diversify research on artificial skin to address complex clinical scenarios. We posit that with continuous advancements and innovations in 3D bioprinting technology and novel biomaterials, artificial skin will become progressively more sophisticated and efficient, thereby enhancing its role in clinical wound management.

## Conclusion

To summarize, we have successfully developed a methodology for whole-layer skin regeneration by integrating tissue engineering with 3D bioprinting technologies. We selected multicomponent photosensitive hydrogels, characterized by excellent printability, low solubility and swelling rates, and stable mechanical properties, to encapsulate Epi-SCs and SKPs for the preparation of a customizable artificial skin via 3D bioprinting. The artificial skin not only facilitates scar-free healing but, more importantly, regenerates skin appendages such as hair follicles, blood vessels, and sebaceous glands. This approach holds significant potential for widespread application in the field of skin tissue engineering and related areas.

## Figures and Tables

**Table 1:** Preparation concentration of multicomponent hydrogel

Concentration (w/v)	GelMA (g)	HAMA (g)	LAP (g)
5% GelMA-1% HAMA	0.5	0.1	0.002
5% GelMA-0.5% HAMA	0.5	0.05	0.002
5% GelMA-0.1% HAMA	0.5	0.01	0.002
5% GelMA	0.5	0	0.002
10% GelMA-1% HAMA	1	0.1	0.002
10% GelMA	1	0	0.002
1% HAMA	0	0.1	0.002

**Table 2:** The primers used for murine gene amplification

Gene	Forward	Reverse
*GAPDH*	AGGTCGGTGTGAACGGATTTG	TGTAGACCATGTAGTTGAGGTCA
*Nanog*	TGTGCACTCAAGGACAGGTT	GGTGCTGAGCCCTTCTGAATC
*Oct4*	CGGAAGAGAAAGCGAACTAGC	ATTGGCGATGTGAGTGATCTG
*c-Myc*	ATGCCCCTCAACGTGAACTTC	CGCAACATAGGATGGAGAGCA
*Sox2*	TCCATGGGCTCTGTGGTCAAG	TGATCATGTCCCGGAGGTCC
*Fibronectin*	ATGTGGACCCCTCCTGATAGT	GCCCAGTGATTTCAGCAAAGG
*a-SMA*	TGAGCAACTTGGACAGCAACA	CTTCTTCCGGGGCTCCTTATC
*Bmp4*	CAGGGAACCGGGCTTGAG	CTGGGATGCTGCTGAGGTTG
*Collagen I*	GCTCCTCTTAGGGGCCACT	CCACGTCTCACCATTGGGG
*Nestin*	GGTTCCCAAAGAGGTGTCCG	CAGCAAACCCATCAGACTCCC
*PDGF-a*	ACGCATGCGGGTGGACTC	GATACCCGGAGCGTGTCAGTTAC
*Akp2*	TCGGAACAACCTGACTGACCC	CTGCTTGGCCTTACCCTCATG

**Table 3:** Application of diverse stem cell types in HFs Regeneration

Stem cells	The signaling pathway of HFs regeneration	Markers	Advantages	Disadvantages	Reference
DPCs	Wnt/β-catenin, SHH, NF-κB, JAK-STAT	ALP, α-SMA, Versican, Corin, CD133, β-catenin	Directly affects the process of hair follicle regeneration	Difficulty of access	66-73
iPSCs	TGF-β/BMP, and FGF	Nanog, Oct4, SOX2, c-Myc, KLF4	Personalization available	Potential tumour-causing risks	74-76
SKPs	PI3K, MAPK	Nestin, fibronectin, BMP6, SOX2, OCT4, CD200, CD73, CD90, CD105, CD271, CD133, p63, K15, K19, SSEA-4	Maintenance of cellular properties *in vitro*	Age limits of sources	77-81
Epi-SCs	PI3K/Akt, Wht/β-catenin, SHH, Notch, BMP	CD29, CD49f, CK5, CK14	Anti-keloidal effects, potential regenerative capacity of appendages, ease of access, wide range of sources	Potential biosafety issue in clinic: *in vitro* residues	82-90
HFSCs	Wnt/β-catenin, Hedgehog, Notch, TGF-β/BMP	CK19, CD15, CD200, Lgr5	Remodel the skin microenvironment	Difficulty in cell identification and mass culture	91-95
MSCs	Wnt/β-catenin, BMP, NF-κB, JAK/STAT	CD105, CD90, CD73, CD44, CD13, CD29, CD133, CD27	Rich sources, ease of access, important immunomodulatory activity	Clinical side-effects unknown	96-99
